# Follistatin-like protein 1 (FSTL1) modulates bone remodeling and attenuates bone loss in a mouse model of postmenopausal osteoporosis

**DOI:** 10.1093/jbmrpl/ziag037

**Published:** 2026-03-12

**Authors:** Hao Yuan, Bao-Rui Chen, Fu-Xing Han, Kai Huang, Zhi-Hao Jia, Ying-Ying Dong, Lin Bo

**Affiliations:** Department of Rheumatology, The Second Affiliated Hospital of Soochow University, Suzhou 215004, China; Department of Rheumatology, The Second Affiliated Hospital of Soochow University, Suzhou 215004, China; CAM-SU Genomic Resource Center, Soochow University, Suzhou, China; CAM-SU Genomic Resource Center, Soochow University, Suzhou, China; Cambridge-Suda Genomic Resource Center, Suzhou Medical College, Soochow University, Suzhou 215000, China; CAM-SU Genomic Resource Center, Soochow University, Suzhou, China; Department of Rheumatology, The Second Affiliated Hospital of Soochow University, Suzhou 215004, China

**Keywords:** FSTL1, postmenopausal osteoporosis, osteoblasts, osteoclasts, Menopause

## Abstract

This study systematically delineates the expression dynamics of follistatin-like protein 1 (FSTL1) in postmenopausal osteoporosis (PMOP) and elucidates its role in skeletal homeostasis. Integrated analyses of clinical serum samples and the GTEx database revealed a pronounced reduction in circulating FSTL1 levels in PMOP patients. In vivo, Fstl1^+/−^ ovariectomized mice displayed exacerbated bone loss—evidenced by diminished BMD and trabecular volume—together with impaired osteoblastic activity and heightened osteoclast activation. Complementary in vitro experiments showed that FSTL1 over-expression markedly enhanced osteogenic differentiation, as reflected by increased alkaline phosphatase activity and matrix mineralization, whereas FSTL1 knockdown produced the opposite effect. Conversely, FSTL1 over-expression suppressed osteoclastogenesis, while its silencing facilitated the formation of tartrate-resistant acid phosphatase-positive osteoclasts. Therapeutic administration of recombinant FSTL1 protein in vivo significantly attenuated OVX-induced bone loss and improved trabecular architecture, stimulated osteoblast differentiation, and curtailed osteoclast function. Collectively, these findings position FSTL1 as a down-regulated mediator in PMOP that modulates bone remodeling by simultaneously promoting osteogenesis and restraining osteoclastogenesis, supporting a potential association of FSTL1 with PMOP and its possible relevance as a biomarker and therapeutic target.

## Introduction

Follistatin-like protein 1 (FSTL1) is a secreted glycoprotein originally identified as a mesenchymal-derived factor. It was first isolated from the murine osteoblastic cell line MC3T3-E1 after stimulation with transforming growth factor-β (TGF-β). Subsequent studies have revealed that FSTL1 is expressed across multiple tissues and cell types and exerts pleiotropic regulatory functions in diverse physiological and pathological processes, including embryonic development and disease progression.^1–3^ FSTL1 harbors follistatin-like domains that enable it to interact with a broad repertoire of cytokines and growth factors, suggesting a regulatory role in tissue remodeling. Genetic studies corroborate its physiological importance: global deletion of Fstl1 in mice precipitates perinatal lethality owing to severe respiratory distress and is accompanied by a constellation of skeletal malformations.^4–8^ Sylva and colleagues, using Fstl1^+/−^GFP reporter mice (Fstl1^G/+^), demonstrated that Fstl1 is indispensable for murine embryonic skeletogenesis at E12.5 and E15.5.^6^ Taken together, these observations implicate FSTL1 as a prospective regulator of skeletal physiology. Consistently, across a spectrum of orthopedic disease models, FSTL1 exhibits pronounced shifts in expression coupled with discernible functional consequences. Loss of FSTL1 disrupts TGF-β/Smad3-p38-Akt signaling, constrains the proliferation of embryonic vertebral chondrocytes and diminishes the synthesis of type II collagen and proteoglycans, thereby precipitating widespread skeletal malformations—findings that establish FSTL1 as an indispensable guardian of chondrogenesis and joint homeostasis.^5^ In both rheumatoid and osteoarthritic settings, exogenous FSTL1 administration suppresses the expression of pro-inflammatory (IL-6) and matrix-degradative (MMP-3) factors, markedly attenuating synovitis and cartilage erosion in anti-type II collagen antibody-induced arthritic mice.^9^ Emerging evidence indicates that FSTL1 safeguards joint homeostasis by sustaining cartilage-matrix biosynthesis while curbing terminal chondrocyte differentiation, thereby exerting context-dependent anti-inflammatory and tissue-protective effects.^10^ Collectively, these findings nominate FSTL1 as an inflammatory mediator that forges crosstalk between the innate and adaptive immune compartments. Beyond its immunomodulatory role, emerging reports implicate FSTL1 in tissue-repair programs, such as fracture consolidation, wherein its capacity to drive fibroblast migration and angiogenic sprouting accelerates wound resolution.^11^ Given FSTL1’s role in inflammation-induced osteoclast differentiation, its impact on embryonic bone formation, and its ability to counter classical BMP signaling, we speculate that FSTL1 may regulate bone-metabolic homeostasis.

Postmenopausal osteoporosis (PMOP) is a systemic skeletal disorder driven by estrogen deficiency, which disrupts the balance of bone remodeling and leads to reduced bone mass, micro-architectural deterioration, and increased fracture risk.^12–14^ Globally, over 200 million women are affected.^15^ Current clinical management of PMOP relies chiefly on 2 pharmacological classes: anti-resorptive agents and osteo-anabolic therapies.^16^^,^^17^ Antiresorptives—such as bisphosphonates, SERMs, and the anti-RANKL antibody denosumab—suppress osteoclast-mediated bone loss,^16^^,^^18–20^ whereas anabolic agents, including teriparatide and the anti-sclerostin antibody romosozumab, stimulate osteoblast-driven bone formation.^21^^,^^22^ Although these drugs improve BMD and reduce fracture incidence, their use is limited by safety concerns, treatment-specific adverse effects, and rebound bone loss upon discontinuation, particularly in denosumab-treated patients.^16^^,^^23^^,^^24^ Consequently, there is an urgent need to identify new therapeutic targets that can yield more effective, durable, and safer treatments for PMOP. The prominent involvement of FSTL1 across multiple skeletal disorders has therefore drawn our attention.

This study is the first to establish a significant association between FSTL1 and PMOP and to reveal FSTL1 as a previously unreported regulator of bone homeostasis that concurrently promotes osteogenesis and suppresses osteoclastogenesis. Specifically, FSTL1 expression is markedly lower in the serum and peripheral blood leukocytes of PMOP patients; in an ovariectomized model, Fstl1-haploinsufficient mice display more severe bone loss, whereas systemic administration of recombinant FSTL1 protein substantially restores BMD and trabecular micro-architecture. In vivo and in vitro functional assays further demonstrate that FSTL1 enhances early and late osteoblastic differentiation markers while inhibiting RANKL-induced osteoclast multinucleation and the expression of osteoclastic genes (Ctsk, Nfatc1, and Trap). Prior studies suggest that FSTL1 may interact with the TGF-β/Smad and RANKL/NF-κB signaling pathways involved in bone metabolism and, by alleviating inflammation, restrict osteoclast activation—thereby providing direction for future mechanistic research. Collectively, these findings indicate that FSTL1 is a promising diagnostic biomarker for PMOP and a potential therapeutic target for regulating bone metabolism, offering prospects for safer and more effective anti-osteoporotic interventions.

## Materials and methods

### Animals

All animal experiments were approved by the Ethics Committee of Soochow University (approval no. ZJ-2021-1) and complied with institutional and national guidelines. Heterozygous Fstl1 KO mice (Fstl1^+/−^; targeted allele information provided by the Laboratory Animal Center of Soochow University), together with WT littermates on a C57BL/6J background, were obtained from the Laboratory Animal Center of Soochow University. Female mice aged 6-8 wk were housed under specific-pathogen-free conditions (22-24 °C; 12-h light/dark cycle) in individually ventilated cages (4-5 mice per cage; 32 × 20 × 18 cm) with ad libitum access to standard chow (Suzhou Xinuosai Biotechnology Co., Ltd.) and water. Group allocation was randomized, and investigators responsible for micro-CT, histological quantification, and cell counting were blinded to group identity throughout data collection and analysis. No animals were excluded from analysis. Sample sizes were based on previous studies using OVX and gene-haploinsufficient mouse models and were sufficient to detect biologically meaningful differences in bone phenotypes.

### Human subjects

Serum and peripheral blood leukocyte samples were obtained from patients in the Department of Orthopedics, The Second Affiliated Hospital of Soochow University. The study protocol was approved by the hospital’s Ethics Committee (approval no. 81802194), and written informed consent was obtained from all participants and their guardians. Between June 2019 and November 2020, peripheral blood was collected after an overnight fast. All participants included in the serum and peripheral blood analyses were female. Based on BMD measurements, participants were categorized into normal, osteopenia, and osteoporosis groups. The harvested serum and leukocytes were used for subsequent assays of bone-metabolism markers and gene-expression analyses. Baseline demographic and clinical characteristics of the study population, including age, sex, menopausal status, and group allocation, are summarized in Table 1.

**Table 1:** Fstl1 primer sequence list.

**Gene**	**Primer sequences (5′→3′)**
Fstl1-forward	CTCCCACCTTCGCCTTAAC
Fstl1-reverse	CGGCTAGGAAAGACTTGGAA

In addition, we conducted a secondary analysis of the adult transcriptome data released by the GTEx Portal (Release V10). Using the cross-tissue TPM matrix “GTEx Analysis v10 RNASeQCv2.4.2 gene tpm.gct.gz” and its clinical annotation file, we selected “Whole Blood” samples and excluded records lacking age or sex, yielding 755 individuals (493 men and 262 women, aged 20-79 yr). Within R 4.2.2, we extracted FSTL1 (ENSG00000163430) expression, set TPM values <0.1 to 0, and transformed the data as log₂(TPM + 1) for subsequent analyses. Comparisons were performed as follows: (1) by sex (male vs female); (2) by age thresholds (<50 yr/≥50 yr and <70 yr/≥70 yr); and (3) by decade (20-29, 30-39, 40-49, 50-59, 60-69, and 70-79).

### Real-time qPCR

Total RNA was extracted from patient specimens, mouse bone tissue, and MC3T3-E1 cells with TRIzol reagent (Takara) according to the manufacturer’s instructions. After measuring concentration and purity, 1 μg of RNA was reverse-transcribed into complementary DNA (cDNA) using the PrimeScript RT Reagent Kit (Takara). Real-time quantitative PCR was performed on an ABI CFX-Connect Real-Time PCR System with SYBR Premix Ex Taq II (Takara) prepared as specified by the supplier. A melting-curve analysis was automatically generated at the end of each run to verify amplification specificity. Primer sequences for Fstl1 are provided in the table below; Gapdh served as housekeeping genes. Relative expression levels were calculated using the 2^−ΔΔCt method. All samples were analyzed in triplicate, and results are expressed as the mean ± SD.

### Osteoblast culture and osteogenic induction

In vitro osteogenic assays were performed with the murine pre-osteoblastic cell line MC3T3-E1. Frozen cells were rapidly thawed in a 37 °C water bath, mixed 1:1 with α-MEM (HyClone) complete medium containing 10% FBS (Gibco) and 1% penicillin-streptomycin (Gibco), and centrifuged at 118g to remove cryoprotectant. The pellet was resuspended and seeded into culture flasks, then incubated overnight at 37 °C in 5% CO₂ to allow attachment. When cultures reached ~80% confluence, the growth medium was replaced with osteogenic induction medium—complete α-MEM supplemented with 10 mM β-glycerophosphate (Sigma, G9422) and 50 μg/mL ascorbic acid (Sigma, A5960). The induction medium was refreshed every 2 d. Cells were harvested or fixed at defined time points (eg, day 7 and day 21) for alkaline phosphatase (ALP) activity assays and Alizarin Red S (ARS) staining to evaluate osteogenic differentiation and matrix mineralization.

### Osteoclast culture and differentiation

Bone-marrow-derived macrophage (BMM) assays were employed. Bone marrow cells were flushed from mouse femora and tibiae with α-MEM and seeded in α-MEM supplemented with 10% FBS and 1% penicillin-streptomycin. After overnight incubation at 37 °C in 5% CO₂ to permit adherence, non-adherent cells were collected and cultured for 3 d in α-MEM containing 30 ng/mL M-CSF (R&D Systems) to generate BMMs. BMMs were then plated in 96-well plates at 1 × 10^4^ cells per well and incubated for 5-7 d in α-MEM supplemented with 30 ng/mL M-CSF and 50 ng/mL RANKL (R&D Systems) to induce osteoclast differentiation.

### TRAP staining and bone resorption assay

Decalcified, paraffin-embedded bone sections were processed for tartrate-resistant acid phosphatase (TRAP) histochemistry using a commercial TRAP staining kit (387A-1KT, Sigma) by the manufacturer’s protocol. Sections were deparaffinized, rehydrated, and incubated with the TRAP substrate solution, and color-developed as directed. Multinucleated cells exhibiting a purplish-red reaction and containing 3 or more nuclei were classified as TRAP-positive osteoclasts under light microscopy. Staining area and optical density were quantified with ImageJ software, and results from 3 independent experiments are expressed as the mean ± SD.

For the bone resorption assay, mature osteoclasts were cultured on Corning Osteo Assay plates. Following incubation, cells were removed via ultrasonication to reveal the resorption pits, which were then imaged and analyzed using SEM.

### ALP staining and alizarin red S (ARS) staining

MC3T3-E1 cells were cultured in six-well plates and induced to undergo osteogenic differentiation. On day 7, ALP staining was performed with a BCIP/NBT ALP color-development kit (Beyotime Biotechnology) according to the manufacturer’s instructions: after removing the medium and gently rinsing with PBS, working solution was added, and the plates were incubated at 37 °C until a purple-black precipitate appeared, whereupon the reaction was stopped. Following staining, cultures were photographed under an optical microscope, and, using the same kit’s extraction method, absorbance at 405 nm was measured to calculate ALP activity as an early osteogenic marker. On day 21, ARS staining was carried out with an Alizarin Red S calcium-staining kit (Beyotime Biotechnology): after fixation, working solution was applied, and the cells were incubated for 30 min at room temperature in the dark, and the reaction was terminated once red mineralized nodules became visible. After thorough PBS washes, bound dye was dissolved with 10% cetyltrimethylammonium bromide and quantified at 562 nm to assess matrix mineralization. All micrographs were analyzed with ImageJ to determine staining area and integrated density, and quantitative data are presented as the mean ± SD from 3 independent experiments.

### Western blot

Self-prepared RIPA lysis buffer (supplemented with 1% phosphatase inhibitor and 1% protease inhibitor PI, Roche) was added to PBS-washed tissues or cells, and the suspension was lysed on ice; the lysate was centrifuged at 12 000 × *g* for 15 min at 4 °C, the supernatant was collected, and the protein concentration was measured with a BCA kit (Beyotime Biotechnology). An aliquot of 30-50 μg protein was mixed with 5 × SDS-PAGE loading buffer, denatured at 100 °C for 5 min, separated on a 10% SDS-PAGE gel, and transferred to a PVDF membrane. The membrane was blocked with 5% non-fat milk (Suzhou Yimingda Biotechnology) for 1 h at room temperature, incubated overnight at 4 °C with the respective primary antibody, washed 3 times for 5 min each with TBST, and then incubated for 1 h at room temperature with HRP-conjugated secondary antibody (Proteintech, 1:5000). Antibodies against RUNX2, SP7, NFATC1, and CTSK were purchased from Abcam, while the anti-GAPDH antibody and anti-FSTL1 antibody were obtained from Proteintech. Signals were developed with ECL chemiluminescent reagent (Novezan), captured using a Shanghai Tianneng imaging system, and quantified with ImageJ; relative expression levels were normalized to GAPDH.

### Fstl1 overexpression and knockdown

Fstl1 gain- and loss-of-function experiments were performed in MC3T3-E1 pre-osteoblastic cells and BMMs. Cells were infected with lentiviral vectors encoding murine Fstl1 (Fstl1-OE) or short hairpin RNAs targeting Fstl1 (shFstl1). Empty vector and scrambled shRNA lentiviruses served as controls. Lentiviral transduction was conducted at an MOI of 10-20 in the presence of polybrene (8 μg/mL), and the medium was replaced after 24 h. Infected cells were maintained for an additional 48-72 h before harvesting for expression analysis. Fstl1 mRNA and protein levels were quantified by qPCR and Western blotting, respectively, to confirm overexpression or knockdown efficiency.

### OVX model and treatment

A PMOP mouse model was generated by randomly assigning equal numbers of Fstl1^+/−^ and WT mice to either bilateral ovariectomy (OVX) or Sham surgery groups. OVX mice underwent removal of both ovaries under isoflurane anesthesia, whereas Sham mice received an identical incision without ovary removal. After surgery, mice were maintained for 8 wk to establish the bone-loss phenotype characteristic of PMOP. All surgical procedures and postoperative monitoring were performed by investigators blinded to genotype and treatment group.

As for recombinant FSTL1 treatment, recombinant murine FSTL1 protein (PeproTech) was administered to OVX mice immediately after surgery by intraperitoneal injection at a dose of 100 μg/kg body weight, 3 times per week for 8 wk. Control mice received an equivalent volume of vehicle on the same schedule.

### Micro-CT analysis

Mouse femora fixed in 4% paraformaldehyde for ≥24 h were rinsed twice in PBS, mounted on the sample stage of a micro-CT system (SkyScan 1176), and scanned at 50 kV and 200 μA. Two-dimensional reconstruction was first performed on the acquired images; after rotational alignment in Dataview, the reference layer was set in CTan by identifying the protrusion of the femoral growth plate, from which a position 100 slices proximally along the diaphysis was designated as the starting layer for cancellous-bone analysis. A further 150 slices in the same direction constituted the region of interest for cancellous-bone microstructural assessment, followed by three-dimensional reconstruction in Mimics. Cortical-bone parameters were evaluated in a region extending an additional 300 slices proximally beyond the cancellous-bone ROI, and the same software was used for 3-D reconstruction and microstructural quantification.

### Immunohistochemistry

Paraffin sections of the distal femoral metaphysis were deparaffinized in xylene and rehydrated through a graded ethanol series. Target regions were circled and covered with a pancreatin antigen-retrieval solution, incubated at 37 °C for 10 min, and rinsed 3 times in distilled water; endogenous peroxidase was blocked with 3% H₂O₂ for 10 min at room temperature, followed by 3 additional washes. Sections were then blocked with 5% goat serum for 1 h at room temperature and incubated with anti-OCN primary antibody (Abcam, #ab93876) overnight at 4 °C (or 1 h at room temperature); after 3 washes, an HRP-conjugated polymer secondary antibody (Proteintech) was applied for 1 h at room temperature, and the sections were washed 3 more times. Chromogenic development with DAB for 1-2 min was stopped when an appropriate brown signal appeared, after which the slides were counterstained with hematoxylin and blued under running tap water. Finally, the sections were dehydrated through 75%-100% ethanol, cleared in xylene, mounted with neutral resin, and observed and photographed under a light microscope.

### Enzyme-linked immunosorbent assay

Fasting venous blood was collected from participants in the early morning, allowed to clot for about 30 min at room temperature, and centrifuged at 3500 × *g* for 10 min to obtain serum, which was stored at −80 °C until use. Serum FSTL1 levels were determined with a commercial FSTL1 ELISA kit (R&D Systems). Following the kit instructions, standards and working reagents were prepared, samples or standards were added to 96-well plates pre-coated with capture antibody, incubated at room temperature, washed thoroughly, and then incubated with HRP-conjugated secondary antibody; substrate solution was added for color development. After the reaction was stopped, absorbance was read at the specified wavelength on a SpectraMax multimode plate reader (Molecular Devices), and FSTL1 concentrations were calculated from the standard curve. All samples were assayed in duplicate, and the results are expressed as mean ± SD.

### Statistical analysis

All results are presented as mean ± SD. Biological replicates were performed unless otherwise specified. A 2-tailed Student’s *t*-test was used for comparisons between 2 groups, while one-way ANOVA followed by Tukey’s post hoc test was applied for comparisons involving more than 2 groups. Statistical analyses were conducted using GraphPad Prism (version 9.0). For animal survival analysis, differences were evaluated using the log-rank test. Statistical significance was defined as ^****^p < .0001; ^***^p < .005; ^**^p < .01; ^*^p < .05. All experiments were performed in at least triplicate.

## Results

### FSTL1 is significantly down-regulated in the PMOP population

**Figure 1:**
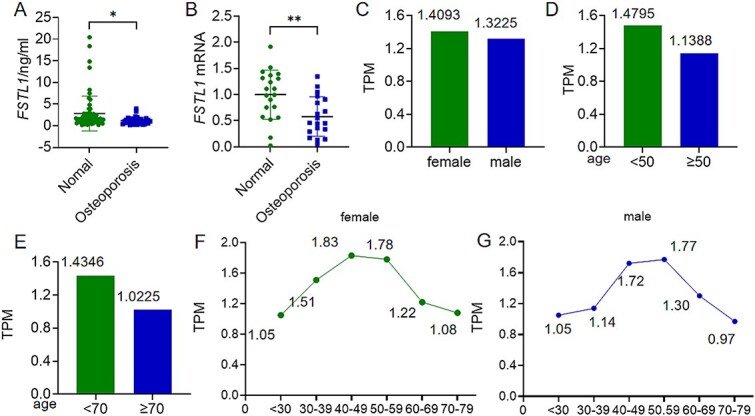
Age-associated down-regulation of FSTL1. (A) Serum FSTL1 concentrations in the normal and osteoporosis groups measured by ELISA. (B) Relative FSTL1 mRNA expression in peripheral blood leukocytes from the 2 groups determined by qPCR. (C) GTEx analysis of sex differences in blood-cell FSTL1 expression. (D) GTEx comparison of FSTL1 expression in individuals aged <50 yr and ≥50 yr. (E) GTEx comparison of FSTL1 expression in individuals aged <70 yr and ≥70 yr. (F) Age-related trend of blood-cell FSTL1 expression in men. (G) Age-related trend of blood-cell FSTL1 expression in women. Data are expressed as mean ± SD; 2-group comparisons were performed with 2-tailed unpaired Student’s *t*-tests. ^*^p < .05, ^**^p < .01, ns denotes no significant difference. Transcripts per million (TPM): this is the most used standardized unit in RNA sequencing (RNA-seq) data. It represents the proportion of a certain gene’s transcripts among all transcripts in a specific sample or tissue. After standardization, it enables comparability between different samples and genes.

FSTL1 is widely recognized as a secreted glycoprotein with regulatory functions across multiple pathological conditions and can be detected in the circulation. Against this background, to explore how FSTL1 expression relates to sex and age, we collected peripheral blood from participants after obtaining informed consent. Based on BMD testing, samples were stratified into Normal and Osteoporosis groups; serum FSTL1 protein was measured by ELISA, and relative FSTL1 mRNA in peripheral blood leukocytes was quantified by real-time qPCR (Figure 1A and B). Compared with the Normal group, the Osteoporosis group showed a significant reduction in both serum FSTL1 protein and leukocyte FSTL1 mRNA, indicating down-regulation of FSTL1 in osteoporotic subjects (Figure 1A and B). We next interrogated the GTEx database (https://gtexportal.org/home/) and found no sex-related difference in blood-cell FSTL1 expression (Figure 1C); however, expression was lower in individuals ≥50 yr than in those <50 yr (*p* < .05) and even further reduced in those ≥70 yr compared with <70 yr (*p* < .05) (Figure 1D and E). In both sexes, FSTL1 expression rose gradually before 40 yr, peaked between 40 and 60 yr, and declined after 60 yr (Figure 1F and G). Taken together, the down-regulation of FSTL1 in postmenopausal women may mirror accelerated bone loss and heightened osteoporosis risk, lending mechanistic support for FSTL1 as both a biomarker and a potential therapeutic target in PMOP.

### Fstl1 exacerbates OVX-induced bone loss by modulating osteoblastic and osteoclastic activity

**Figure 2:**
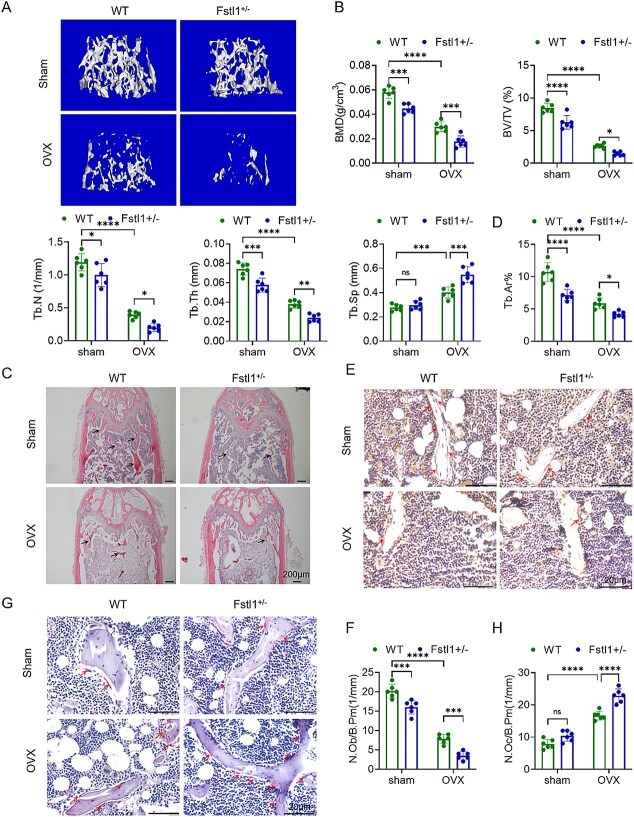
Fstl1 haploinsufficiency disrupts bone homeostasis and aggravates OVX-induced bone loss. (A) Three-dimensional micro-CT reconstructions of femora. (B) quantitative micro-CT results. (C) Representative H&E images of distal femoral trabeculae. (D) Quantitative analysis of trabecular area (Tb.Ar) from H&E sections. (E) Osteocalcin (OCN) immunohistochemistry. (F) Quantitative analysis of osteoblast number per bone perimeter (N.Ob/B.Pm, 1/mm) in the indicated experimental groups. (G) TRAP staining depicting osteoclast distribution (red arrows). (H) Quantitative analysis of osteoclast number per bone perimeter (N.Oc/B.Pm, 1/mm) in the indicated experimental groups. Sample sizes: WT-sham (*n* = 6), Fstl1^+/−^ sham (*n* = 6), WT-OVX (*n* = 6), and Fstl1^+/−^ OVX (*n* = 6). Data are expressed as mean ± SD; group comparisons were conducted with 2-way ANOVA. ^*^p < .05, ^**^p < .01, ^***^p < .001, ^****^p < .0001; ns, not significant.

As homozygous Fstl1 KO mice (Fstl1^−/−^) die within hours of birth from respiratory distress,^4^^,^^6^ we employed heterozygous mice (Fstl1^+/−^) to evaluate whether Fstl1 influences estrogen-deficiency-induced osteoporosis. Western blot analysis revealed a marked reduction in FSTL1 protein expression in tissues/cells derived from Fstl1^+/−^ mice compared to their WT littermates. (Figure S1A and B). Mice were randomly allocated to 4 groups: WT-Sham, Fstl1^+/−^ Sham, WT-OVX, and Fstl1^+/−^ OVX. Eight weeks after surgery, femora were scanned by micro-CT (Figure 2A). As expected, WT-Sham animals displayed an intact, densely interconnected trabecular network, whereas WT-OVX mice showed pronounced bone loss and trabecular deterioration, confirming successful model establishment (Figure 2A). The Fstl1^+/−^ Sham group exhibited a slight reduction in bone mass relative to WT-Sham, suggesting that Fstl1 may also help maintain skeletal homeostasis under physiological conditions; quantitative metrics supported this observation (Figure 2B). Notably, compared with WT-OVX mice, Fstl1^+/−^ OVX animals showed marked decreases in BMD, bone volume fraction (BV/TV), trabecular thickness (Tb.Th), and trabecular number (Tb.N), together with a significant increase in trabecular separation (Tb.Sp), indicating that loss of Fstl1 aggravates OVX-induced bone depletion (Figure 2B). Detailed histological examination of femora corroborated the micro-CT findings. H&E staining revealed trabecular rarefaction and structural degradation in Fstl1^+/−^ Sham mice relative to WT-Sham, with similar changes observed between the WT-OVX and Fstl1^+/−^ OVX groups (Figure 2C). Quantitative assessment mirrored the staining results: compared with WT-Sham, the trabecular area (Tb.Ar) was significantly reduced in Fstl1^+/−^ Sham mice, and a comparable decline was evident between WT-OVX and Fstl1^+/−^ OVX cohorts (Figure 2D). Collectively, these data indicate that Fstl1 contributes to both age-related and PMOP.

To elucidate the mechanism by which Fstl1 deficiency precipitates bone loss, femoral sections from the above-described models were subjected to osteocalcin (OCN) immunohistochemistry to label osteoblasts and, in parallel, TRAP staining to assess osteoclast activity, thereby determining whether haploinsufficiency of Fstl1 perturbs bone-formation-bone-resorption balance. Osteocalcin staining revealed fewer OCN^+^ osteoblasts along the trabecular surfaces of Fstl1^+/−^ Sham mice than in WT-Sham controls, with a further reduction in the Fstl1^+/−^ OVX group relative to WT-OVX (Figure 2E). Quantitative analysis confirmed a significant decrease in the number of osteoblasts per bone perimeter (N.Ob/B.Pm, 1/mm) in Fstl1^+/−^ Sham vs WT-Sham and significantly lower in Fstl1^+/−^ OVX vs WT-OVX, indicating that Fstl1 haploinsufficiency per se weakens osteogenic activity and exacerbates the estrogen-deficiency-induced decline in osteoblast numbers (Figure 2F). Tartrate-resistant acid phosphatase staining demonstrated no significant difference in the number of TRAP^+^ osteoclasts lining trabeculae between Fstl1^+/−^ Sham and WT-Sham mice, whereas Fstl1^+/−^ OVX animals displayed a pronounced increase compared with WT-OVX (Figure 2G). Quantitative analysis confirmed that Fstl1 haploinsufficiency alone did not appreciably alter osteoclast numbers, but under OVX conditions, it significantly elevated the abundance of trabecular TRAP^+^ osteoclasts, suggesting that Fstl1 modulates bone resorption by regulating osteoclast activity in mice (Figure 2H). Estrogen deficiency is known to enhance osteoclastogenesis through increased RANKL signaling and inflammatory cues. Our data suggest that Fstl1 haploinsufficiency may further sensitize osteoclast-lineage cells to the OVX microenvironment, thereby exacerbating osteoclast expansion and bone resorption under estrogen-deficient conditions.

### Fstl1 promotes osteoblast differentiation

**Figure 3:**
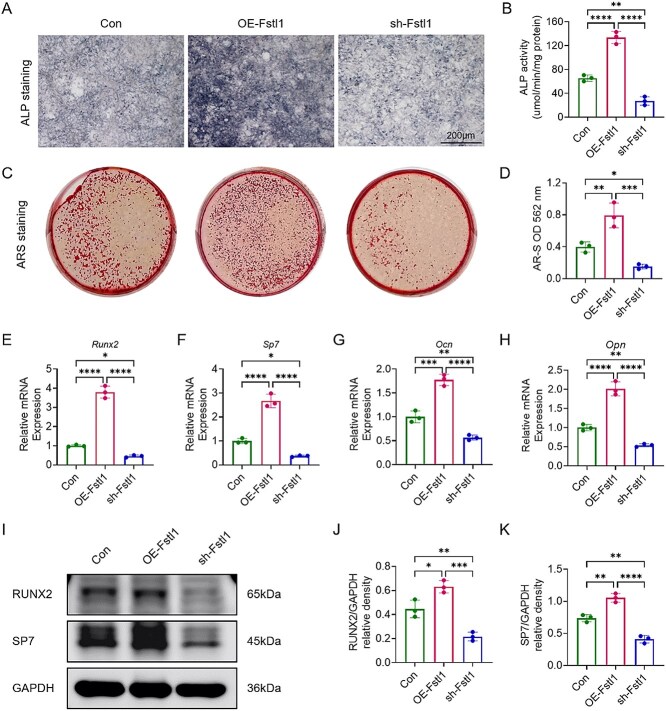
Fstl1 enhances the osteogenic differentiation potential of MC3T3-E1 cells. (A) Representative alkaline-phosphatase (ALP) staining at 7 d. (B) Quantitative ALP activity. (C) Representative Alizarin Red S (ARS) staining at 14 d. (D) Quantification of ARS absorbance (OD_562_). (E-H) Relative mRNA expression of Runx2, Sp7, Ocn, and Opn at 14 d determined by RT-qPCR. (I) Western-blot bands for RUNX2 and SP7. (J and K) Densitometric ratios of RUNX2/GAPDH and SP7/GAPDH. Sample sizes: con (*n* = 3), OE-Fstl1 (*n* = 3), and sh-Fstl1 (*n* = 3). Data are presented as mean ± SD; inter-group comparisons were performed using 1-way ANOVA. ^*^p < .05, ^**^p < .01, ^***^p < .001, ^****^p < .0001; ns, not significant.

Building on the in vivo evidence that Fstl1 haploinsufficiency attenuates bone formation, we next examined its regulatory effect on osteoblasts in vitro. Using the mouse pre-osteoblastic cell line MC3T3-E1, we established a control group (Con), an Fstl1-overexpression group (OE-Fstl1), and an FSTL1-knockdown group (sh-stl1), and subjected them to osteogenic induction for 7 and 21 d, respectively. Before assessing osteogenic outcomes, we confirmed the effectiveness of Fstl1 modulation. qPCR and Western blot analyses demonstrated a robust elevation of Fstl1 expression in OE-Fstl1 cells and a marked reduction in sh-Fstl1 cells relative to Con, thereby validating the gain- and loss-of-function system used for subsequent assays (Figure S2A and B). After 7 d, ALP staining revealed a uniform and markedly intensified purplish-red signal in OE-Fstl1 cells, whereas sh-Fstl1 cells displayed weaker staining relative to Con (Figure 3A); quantitative ALP activity corroborated these observations, showing a significant increase in OE-Fstl1 and a significant decrease in sh-Fstl1 compared with Con (Figure 3B), indicating that up-regulating Fstl1 enhances early osteogenic ALP activity, whereas its knock-down suppresses it. After 21 d, ARS staining demonstrated abundant, dense red mineralized nodules in OE-stl1 cultures, whereas sh-Fstl1 cultures exhibited only sparse, pale-red deposits (Figure 3C); corresponding ARS quantification (OD_562_) showed OE-Fstl1 values significantly higher than Con and sh-Fstl1 values significantly lower (Figure 3D). Collectively, these data indicate that FSTL1 overexpression promotes late-stage mineralized nodule formation, whereas Fstl1 deficiency impedes the mineralization process.

To further delineate the molecular basis of Fstl1-mediated osteogenesis, we quantified the transcripts of Runx2, Sp7, Ocn, and Opn by RT-qPCR after 14 d of induction (Figure 3E-H). Relative to the Con group, OE-Fstl1 exhibited significant up-regulation of all 4 markers, whereas sh-Fstl1 showed significant down-regulation (Figure 3E-H), supporting a positive regulatory role for Fstl1 in osteogenic differentiation. Western blotting confirmed these mRNA trends: compared with Con, OE-Fstl1 displayed pronounced increases in RUNX2 and SP7 protein expression, while sh-Fstl1 showed marked decreases (Figure 3I). Densitometric analysis revealed RUNX2/GAPDH and SP7/GAPDH ratios significantly higher in OE-Fstl1 and significantly lower in sh-Fstl1 vs Con (Figure 3J and K). Collectively, Fstl1 overexpression markedly elevates osteogenic marker protein levels in MC3T3-E1 cells, whereas Fstl1 knockdown suppresses them, indicating that Fstl1 enhances—and its deficiency diminishes—the osteogenic differentiation potential of MC3T3-E1 cells in vitro.

### Fstl1 suppresses osteoclast differentiation

**Figure 4:**
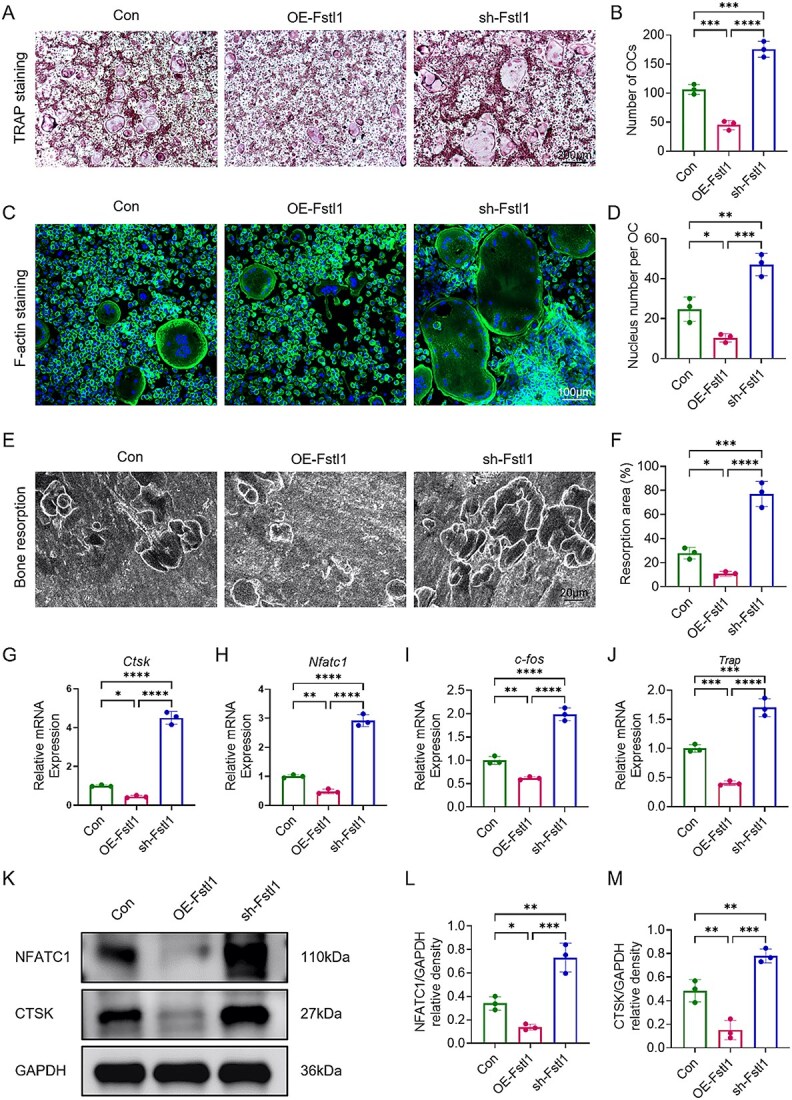
Fstl1 suppresses the osteoclast-differentiation activity of BMMs. (A) TRAP staining showing TRAP^+^ multinucleated osteoclast morphology in each group. (B) Quantitative analysis of TRAP^+^ osteoclast numbers. (C) Dual staining of F-actin rings (green) and DAPI (blue) illustrating the resorptive synapse structure of mature osteoclasts. (D) Average number of nuclei per TRAP^+^ osteoclast. (E and F) Representative images of resorption pits on an osteoassay surface from each group and quantification of the total resorbed area per well (percentage of surface area). (G-J) Relative mRNA expression of Ctsk, Nfatc1, c-Fos, and Trap determined by RT-qPCR. (K) Western-blot bands for CTSK and NFATc1. (L and M) Densitometric ratios of NFATc1/GAPDH and CTSK/GAPDH. Sample sizes: con (*n* = 3), OE-Fstl1 (*n* = 3), and sh-Fstl1 (*n* = 3). Data are presented as mean ± SD; inter-group comparisons were performed with 1-way ANOVA. ^*^p < .05, ^**^p < .01, ^***^p < .001, ^****^p < .0001; ns, not significant.

Postmenopausal osteoporosis is characterized by an increase in osteoclast number and activity. To investigate the role of Fstl1 in osteoclastogenesis, BMMs were first transduced with lentiviruses to generate an Fstl1-overexpression group (OE-Fstl1) and an Fstl1-knockdown group (sh-Fstl1), alongside an unmodified control (Con). Before initiating osteoclast differentiation, we confirmed that lentiviral manipulation effectively altered Fstl1 expression in BMMs (Figure S2C and D). Following transduction, BMMs were cultured for 7 d in osteoclastogenic medium containing 30 ng·mL^−1^ M-CSF and 50 ng·mL^−1^ RANKL. Tartrate-resistant acid phosphatase staining confirmed robust induction in Con cells, which formed numerous dark-purplish TRAP^+^ multinucleated osteoclasts (Figure 4A). Compared with Con, both the number and size of TRAP^+^ osteoclasts were markedly diminished in OE-Fstl1, whereas sh-Fstl1 exhibited a significant increase (Figure 4A), and quantitative counts were concordant: osteoclast numbers were significantly lower in OE-Fstl1 and higher in sh-Fstl1 relative to Con (Figure 4B). Thus, Fstl1 over-expression inhibits, whereas its knock-down promotes, osteoclast formation. Fluorescent staining of filamentous actin (F-actin) visualized the sealing-belt structures of mature osteoclasts, revealing small, sparse rings in OE-Fstl1 and enlarged multinucleated cells with dense F-actin belts in sh-Fstl1 (Figure 4C). Enumeration of nuclei per TRAP^+^ osteoclast showed significantly fewer nuclei in OE-Fstl1 than in Con and significantly more in sh-Fstl1 (Figure 4D), corroborating that Fstl1 over-expression restricts the development of multinucleated osteoclasts. To directly assess osteoclast bone-resorptive function, mature osteoclasts were further subjected to bone resorption assays on bone slices. Compared with control cells, Fstl1 over-expression resulted in a marked reduction in resorption pit area, whereas Fstl1 knock-down significantly increased bone resorption (Figure 4E and F). These results demonstrate that FSTL1 not only suppresses osteoclast differentiation and multinucleation but also attenuates osteoclast-mediated bone-resorptive function.

To clarify the mechanism whereby Fstl1 modulates osteoclast differentiation, the mRNA levels of the osteoclastic markers Ctsk, Nfatc1, c-Fos, and Trap were assessed by RT-qPCR (Figure 4G-J). Relative to the control, all 4 transcripts were markedly down-regulated in the OE-Fstl1 group, whereas they were significantly up-regulated in the sh-Fstl1 group, indicating that Fstl1 suppresses osteoclast formation by negatively regulating key differentiation factors (Figure 4G-J). Consistently, Western blotting showed reduced CTSK and NFATc1 protein abundance in OE-Fstl1 cells and elevated levels in sh-Fstl1 cells compared with the control (Figure 4K); densitometric analysis corroborated that these protein changes paralleled the mRNA trends (Figure 4L and M). Collectively, the data demonstrate that, in vitro, Fstl1 attenuates osteoclastogenesis and bone-resorptive function by limiting multinucleation and down-modulating core osteoclastic signaling molecules.

### Exogenous FSTL1 markedly ameliorates the osteoporotic phenotype in OVX mice

**Figure 5:**
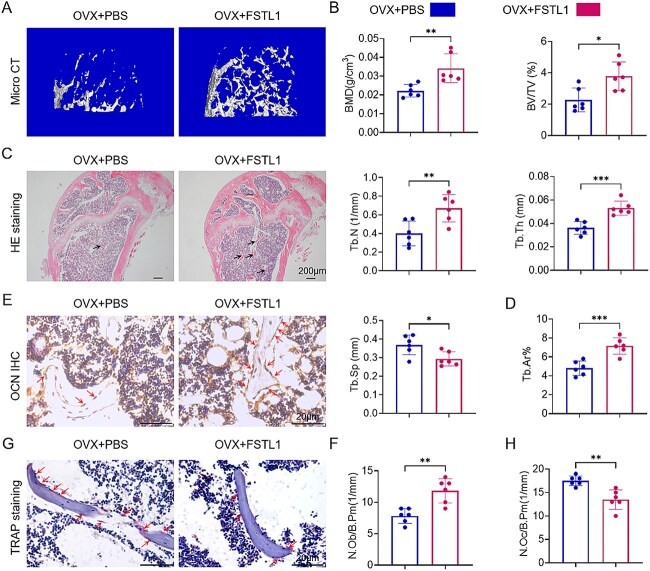
Intraperitoneal administration of FSTL1 markedly restores bone mass in OVX mice. (A) Micro-CT three-dimensional reconstructions showing trabecular morphology in femoral cancellous bone. (B) Quantitative micro-CT parameters (BMD, BV/TV, Tb.Th, Tb.N, and Tb.Sp). (C) H&E staining illustrating trabecular architecture. (D) Quantitative analysis of trabecular area fraction (Tb.Ar%). (E) OCN immunohistochemistry depicting osteoblast distribution (red arrows). (F) Quantitative analysis of osteoblast number per bone perimeter (N.Ob/B.Pm, 1/mm) in the indicated experimental groups. (G) TRAP staining depicting osteoclast distribution (red arrows). (H) Quantitative analysis of osteoclast number per bone perimeter (N.Oc/B.Pm, 1/mm) in the indicated experimental groups. Sample sizes: OVX+PBS (*n* = 6) and OVX+FSTL1 (*n* = 6). Data are expressed as mean ± SD; 2-tailed unpaired Student’s *t*-tests were used for between-group comparisons. ^*^p < .05, ^**^p < .01, ^***^p < .001; ns, not significant.

To assess the influence of FSTL1 on bone architecture after ovariectomy, OVX mice were randomized into an OVX+PBS control group and an OVX+FSTL1 treatment group; the latter received intraperitoneal injections of recombinant FSTL1 (100 μg/kg, three times per week for 8 wk). Micro-CT analysis of femora at week 8 showed that, relative to OVX+PBS, the OVX+FSTL1 group exhibited a denser trabecular network with markedly increased trabecular number and branching (Figure 5A). Quantitative micro-CT data confirmed significant improvements: BMD, bone volume fraction (BV/TV), trabecular thickness (Tb.Th), and trabecular number (Tb.N) were all higher in OVX+FSTL1 mice, whereas trabecular separation (Tb.Sp) was lower, compared with controls (Figure 5B). These findings indicate that exogenous FSTL1 treatment effectively increases bone mass in OVX mice.

Consistent with the micro-CT findings, H&E staining revealed sparse trabeculae with widened inter-trabecular spaces in the OVX+PBS femora, whereas the OVX+FSTL1 femora exhibited compact, thickened trabeculae structures (arrows, Figure 5C). Quantification showed a significantly higher trabecular area fraction (Tb.Ar %) in OVX+FSTL1 mice than in controls, indicating improved micro-architecture (Figure 5D). To further characterize bone cellular changes, osteocalcin (OCN) immunohistochemistry demonstrated a marked increase in OCN^+^ osteoblasts within cancellous bone of the OVX+FSTL1 group (red arrows, Figure 5E); corresponding analysis confirmed a significant increase in the number of osteoblasts per bone perimeter (N.Ob/B.Pm, 1/mm) relative to the OVX+PBS group, suggesting that FSTL1 augments osteoblast abundance and osteogenic marker expression (Figure 5F). In parallel, TRAP staining revealed fewer TRAP^+^ osteoclasts in OVX+FSTL1 femora than in controls (red arrows, Figure 5G), and quantitative data verified a significantly lower percentage of TRAP^+^ cells (Figure 5H). Collectively, these findings demonstrate that exogenous FSTL1 treatment markedly ameliorates the osteoporotic phenotype in OVX mice, as reflected by improved bone mass and micro-architecture accompanied by favorable alterations in bone-cell composition.

## Discussion

FSTL1 is a glycoprotein that regulates development and injury repair in various tissues. The present study supports that FSTL1 exerts a dual role in bone remodeling—enhancing osteoblast-mediated bone formation while suppressing osteoclast-mediated bone resorption. By contributing to the dynamic equilibrium between formation and resorption, FSTL1 may represent a promising therapeutic target for PMOP.

Our data indicate that FSTL1 is associated with enhanced osteogenic capacity. Both in vitro and in vivo analyses revealed increased osteoblast number and osteogenic marker expression following FSTL1 supplementation, consistent with a promotive role in osteoblast differentiation. Although the precise molecular mechanisms were not directly interrogated in this study, existing evidence provides plausible mechanistic links. FSTL1 was originally identified as a TGF-β1-inducible gene in osteoblasts, and its expression is positively regulated by estrogen, suggesting involvement in TGF-β/BMP-related osteogenic regulation.^25^ As a member of the follistatin family, FSTL1 may act similarly: classical follistatin promotes osteogenesis by neutralizing Activin A, which, at late stages of osteoblastogenesis, delays calcification and diminishes mineralized nodule formation—so FSTL1 is likely to exert a pro-osteogenic effect by modulating the balance of TGF-β superfamily ligands.^26^ Moreover, prior studies have demonstrated that FSTL1 can interact with components of the TGF-β receptor complex, including ALK1 and endoglin, thereby affecting downstream Smad2/3 signaling.^10^^,^^27^ In human articular chondrocytes, suppression of FSTL1 attenuates TGF-β-induced Smad3 signaling and precipitates premature hypertrophic differentiation; by analogy in bone, FSTL1 may orchestrate the coupling of bone formation and resorption by modulating osteoblast responsiveness to TGF-β.^5^^,^^10^ Meanwhile, studies have shown that FSTL1, as a TGF-β-induced secreted protein, can enhance Smad3/p38-Akt signaling to promote mesenchymal stem-cell proliferation and drive chondrogenic differentiation; conversely, its deficiency is accompanied by blockade of the TGF-β/Smad3 pathway and embryonic skeletal malformations, suggesting that FSTL1 may facilitate bone formation through TGF-β-related signaling mechanisms.^3^^,^^5^ In summary, FSTL1 promotes bone formation by enhancing osteoblast differentiation and mineralization. It achieves this effect through three coordinated mechanisms: interaction with the TGF-β receptor complex, modulation of TGF-β superfamily ligand activity, and activation of downstream pathways such as Smad2/3-p38-Akt.

In parallel, our results support an inhibitory effect of FSTL1 on osteoclastogenesis. Genetic manipulation of Fstl1 in BMM demonstrated reduced osteoclast differentiation, multinucleation, and bone-resorptive activity in the presence of elevated FSTL1. First, concerning the classical osteoclastogenic signaling axis: RANKL-RANK-NF-κB/NFATc1 in PMOP, the absence of estrogen stimulation leads osteoblasts and osteocytes to up-regulate RANKL and, through paracrine signals, such as complement factor D and C5a, heighten the responsiveness of osteoclast precursors to the RANKL-RANK-NF-κB/NFATc1 pathway. In a murine RA model, FSTL1 exhibits a negative-feedback inhibitory effect: FSTL1 (also termed FRP/TSC-36) is up-regulated by TGF-β in RA synoviocytes and can reduce c-Fos transcript levels within the synovium.^28^ Because c-Fos is indispensable for activating the NFATc1 promoter, the downregulation of c-Fos by FSTL1 indirectly suppresses RANKL-induced NFATc1 activity, thereby disrupting the canonical osteoclastogenic signaling pathway.^9^^,^^28^^,^^29^ Notably, NFATc1 acts as the master switch for osteoclast differentiation; thus, any factor that attenuates this pathway will suppress osteoclastogenesis.^30–33^ In collagen antibody-induced arthritis mice, daily administration of recombinant FSTL1 markedly attenuated joint inflammation and bone destruction; transcriptomic profiling further confirmed that FSTL1 treatment significantly down-regulated c-Fos and its downstream genes (eg, MMP3) within the affected joints.^34^ Consequently, FSTL1 may act as a negative regulator of the RANKL–RANK–NF-κB/NFATc1 axis under inflammatory conditions, thereby suppressing the transcription factors essential for osteoclast differentiation and attenuating both the formation and activity of osteoclasts. Although these pathways were not directly interrogated in the present study, our findings are consistent with this regulatory framework.

Concomitantly, postmenopausal estrogen deficiency rapidly remodels the BM microenvironment: IL-6 and TNF-α released from vascular and stromal cells, together with IL-17 secreted by Th17 lymphocytes, accumulate locally and further drive the NF-κB–NFATc1 cascade, markedly enhancing osteoclast proliferation, fusion and resorptive capacity. Evidence also shows that FSTL1 can down-regulate multiple inflammatory mediators. For example, in arthritic models, FSTL1 treatment significantly reduces IL-6 transcription in synovial tissue, and urinary deoxypyridinoline levels decline in parallel, indicating diminished bone-resorptive activity.^9^ Similarly, in a transplantation-tolerance model, in vivo over-expression of FSTL1 markedly suppressed the production of pro-inflammatory cytokines, including IL-6, IL-17A, and IFN-γ.^34^^,^^35^ IL-17, secreted by Th17 lymphocytes, is a potent osteoclastogenic mediator that induces RANKL expression in osteoblasts; by lowering IL-17A levels, FSTL1 helps curb excessive osteoclast activation.^35^ Moreover, studies indicate that FSTL1 can diminish IL-1β and TNF-α production during organ injury: in a cisplatin-induced renal-damage model, FSTL1 deficiency markedly elevated IL-1β, whereas supplementation with FSTL1 suppressed IL-1β synthesis and alleviated inflammatory injury.^34^ Therefore, under the altered post-menopausal bone-marrow microenvironment, FSTL1 attenuates the local abundance of pro-osteoclastogenic inflammatory mediators by suppressing the production of key bone-resorptive cytokines. This creates conditions that are unfavorable for osteoclast formation and activity. Specifically, FSTL1 directly suppresses osteoclastogenesis by down-regulating the c-Fos–NFATc1 axis and diminishing RANKL–RANK signaling, while indirectly restraining bone resorption by lowering the levels of IL-6, IL-17, and TNF-α and thereby improving the marrow milieu.

The present findings carry important potential clinical implications. First, the marked difference in FSTL1 levels between patient cohorts suggests that serum FSTL1 may serve as a biomarker for osteoporosis: low circulating FSTL1 could indicate an elevated risk of bone loss. Second, our animal studies demonstrate that raising FSTL1 levels restores bone mass in osteoporotic mice, providing preclinical evidence suggesting that FSTL1 is a viable therapeutic target. Unlike most current anti-osteoporotic strategies, which predominantly inhibit resorption or solely stimulate formation, FSTL1 exerts dual effects—promoting osteogenesis and suppressing osteoclastogenesis—thus offering a single intervention capable of correcting both components of dysregulated bone metabolism. Future efforts might focus on developing recombinant FSTL1 protein preparations or small molecules that up-regulate its expression for PMOP therapy and clinical translation.

This study nonetheless has limitations. First, the population-based analysis establishes only an association between FSTL1 and osteoporosis; prospective studies and mechanistic experiments are required to determine whether reduced FSTL1 directly causes bone loss. Second, the use of heterozygous Fstl1-KO mice precludes assessment of complete deficiency, and the experiments were confined to females; tissue-specific deletions and studies in males or other osteoporosis models are warranted to test generalizability. Moreover, mechanistic insights were largely inferred rather than directly measured, as we did not quantify FSTL1-dependent activation of osteoblastic or osteoclastic pathways nor its impact on inflammatory cytokine levels in the bone microenvironment, which should be addressed in future studies. These questions will require further experimentation. Future work should dissect the FSTL1 signaling network through conditional KO or pharmacological inhibition and validate its role in inflammation-linked osteoporosis models. Elucidating FSTL1’s mechanistic contributions to osteoporotic progression may open new avenues for prevention and therapy.

## Supplementary Material

FSTL1_supplementary_materials(1)_ziag037

## Data Availability

The data that support the findings of this study are available from the corresponding author, B.L., upon reasonable request.
