# Supplementary material for: Follistatin-like protein 1 (FSTL1) modulates bone remodeling and attenuates bone loss in a mouse model of postmenopausal osteoporosis
Source: JBMR Plus. 2026 Mar 12;10(5):ziag037. doi: 10.1093/jbmrpl/ziag037 (PMC13050501; doi:10.1093/jbmrpl/ziag037)
Supplement: FSTL1_supplementary_materials(1)_ziag037 [file fstl1_supplementary_materials(1)_ziag037.docx]

# Follistatin-Like Protein 1 (FSTL1) Modulates Bone Remodeling and Attenuates Bone Loss in Postmenopausal Osteoporosis

Hao Yuan^1*^，Bao-Rui Chen^1*^，Fu-Xing Han^2*^，Kai Huang^2^，Zhi-Hao Jia^3^，Ying-Ying Dong^2#†^，Lin Bo^1#^

1.Department of Rheumatology, The Second Affiliated Hospital of Soochow University, Suzhou, 215004 Jiangsu,China

2.CAM-SU Genomic Resource Center, Soochow University，703-3112, 199 Ren-ai Road, Suzhou Industrial Park, Suzhou, Jiangsu, China.

3.Cambridge-Suda Genomic Resource Center, Suzhou Medical College, Soochow University, Suzhou 215000, China.

#Correspondence authors：

Lin Bo (bolin@suda.edu.cn), Department of Rheumatology, The Second Affiliated Hospital ofSoochow University, Suzhou, 215004Jiangsu,China

Ying-Ying Dong(yydong@suda.edu.cn), CAM-SU Genomic Resource Center, Soochow University，703-3112, 199 Ren-ai Road, Suzhou Industrial Park, Suzhou, Jiangsu, China. We note with great sadness that Dr.Ying-Ying Dong passed away during the preparation of this manuscript.

*These authors contributed equally to this work and should be considered co-first authors

*Co-first authors：

Hao Yuan [(1113168915@qq.com)](mailto:(emailA@xxx.edu)), Department of Rheumatology, The Second Affiliated Hospital ofSoochow University, Suzhou, 215004Jiangsu,China

Bao-Rui Chen [(19922807842@163.com)](mailto:(emailB@xxx.edu)), Department of Rheumatology, The Second Affiliated Hospital ofSoochow University, Suzhou, 215004Jiangsu,China

Fu-Xing Han [(15837636370@163.com),](mailto:(15837636370@163.com),) CAM-SU Genomic Resource Center, Soochow University，703-3112, 199 Ren-ai Road, Suzhou Industrial Park, Suzhou, Jiangsu, China.

Supplemental Fig 1. FSTL1 protein expression is significantly reduced in femoral bone tissue of Fstl1⁺/⁻ mice.

Supplemental Fig 2. Validation of FSTL1 overexpression and knockdown in MC3T3 and BMM cells.

Table1.Baseline Characteristics of the Study Participants


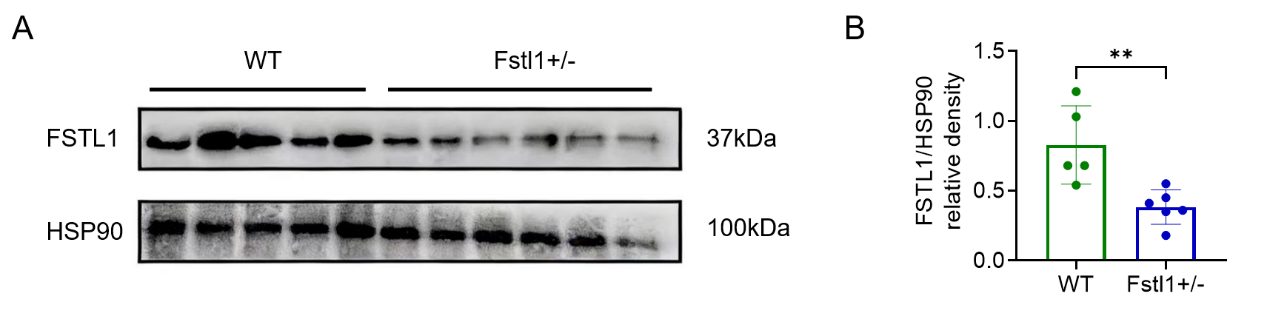


**Supplemental Fig 1.** **FSTL1 protein expression is significantly reduced in femoral bone tissue of Fstl1⁺/⁻ mice.**

(A, B) Representative Western blot images of FSTL1 protein expression in femoral bone tissue lysates from wild-type (WT, n=5) and Fstl1⁺/⁻ mice (n=6). Data are expressed as mean ± SD; two-tailed unpaired Student’s t-tests were used for between-group comparisons. ***p* < 0.01.


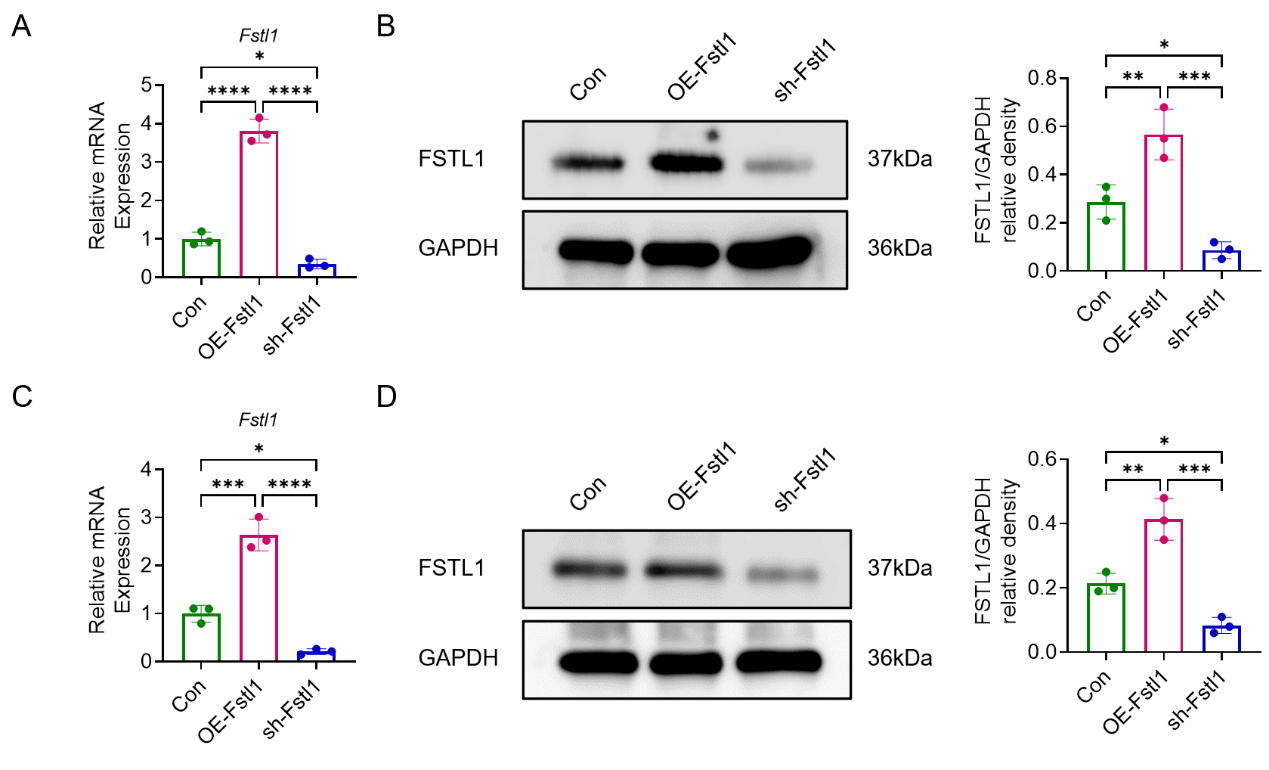


**Supplemental Fig 2.** **Validation of FSTL1 overexpression and knockdown in MC3T3 and BMM cells.**

(A, B) qPCR and western blot analyses confirm the significant overexpression and knockdown, respectively, of Fstl1 in MC3T3 cells (n=3). (C, D) Similarly, the efficient modulation of Fstl1 expression in BMMs is validated at both the mRNA and protein levels (n=3). Data are presented as mean ± SD; inter-group comparisons were performed with one-way ANOVA. **p* < 0.05, ***p* < 0.01, ****p* < 0.001, ****p < 0.0001.

Table1.Baseline Characteristics of the Study Participants

| Characteristic | Normal  (n = 13) | Osteopenia  (n = 17) | Osteoporosis (n = 9) | *P* value |
| --- | --- | --- | --- | --- |
| Age  (years, mean ± SD) | 58.08±16.11 | 70.12±8.63 | 73.56±11.36 | 0.006 |
| Female, n (%) | 13 (100%) | 17 (100%) | 9 (100%) | — |
| Postmenopausal, n (%) | 7 (53.8%) | 17 (100%) | 8 (88.9%) | 0.004 |

Footnotes:

1. P value calculated using one-way analysis of variance (ANOVA).

2. P value calculated using chi-square test.

3. Categorical variables are presented as number (percentage), and continuous variables are presented as mean ± standard deviation.

4. Bone mineral density groups were defined according to T-scores: normal (T ≥ −1.0), osteopenia (−2.5 < T < −1.0), and osteoporosis (T ≤ −2.5).
